# Type I Neurofibromatosis: Case Report and Review of the Literature Focused on Oral and Cutaneous Lesions

**DOI:** 10.3390/dermatopathology8010003

**Published:** 2021-01-07

**Authors:** Samanta Buchholzer, Raùl Verdeja, Tommaso Lombardi

**Affiliations:** Unit of Oral Medicine and Maxillofacial Pathology, Division of Maxillofacial and Oral Surgery, Department of Surgery, Geneva University Hospitals, 1205 Geneva, Switzerland; R.Verdeja@verdeja.ch (R.V.); Tommaso.Lombardi@hcuge.ch (T.L.)

**Keywords:** type 1 neurofibromatosis, oral mucosa, neurofibromas, cutaneous lesions, oral diseases

## Abstract

Neurofibromatosis type 1 (NF1) is a common genetic disease whose dermatological lesions are at the forefront of its development. Cutaneous manifestations include café au lait spots, intertriginous freckling, and neurofibromas which appear during childhood and adolescence and are part of the clinical criteria to diagnose NF1. However, it is only recently that oral manifestations have been highlighted in many studies as frequently associated to NF1. This article aims to review oral and cutaneous manifestations related to NF1 and to report a case of a 51-year-old male with skin and oral neurofibromas related to NF1. Our patient presented with lesions on the gingiva, a rare localization that takes a hypertrophic form mimicking other various pathological conditions. Although not frequent, malignant transformation in NF1, especially regarding plexiform neurofibromas, is well established. Patients with NF1 therefore have regular follow-ups based on clinical examination, as sarcomatous transformation brings an extremely poor prognosis, recurrences and distant metastasis being common.

## 1. Introduction

Neurofibromatosis type 1 (NF1) or von Recklinghausen’s neurofibromatosis is one of the most common autosomal dominant disease [1,2] with a prevalence between 1/2000 and 1/3500 [3,4]. It is caused by the mutation of a tumor suppressor gene NF1 located in the long arm of chromosome 17 (17q11.2). Half of the patients affected by NF1 have a positive family history, while the remaining half represent de novo mutations [3,4]. NF1 has almost 100% penetrance at 5 years of age [1], however the phenotypic expression is extremely variable even for NF1 patients within the same family [5]. NF1 has no prevalence for gender or race [5].

NF1 usually presents with café au lait spots, intertriginous freckling, Lisch nodules of the iris, cutaneous neurofibroma, and osseous dysplasia including decreased bone mineral density, scoliosis, and pseudoarthrosis. Other features of NF1 are mediastinal, spinal, and peripheral nerve neurofibromas, central nervous system tumors such as optic glioma, oral and maxillofacial abnormalities, neurologic or cognitive impairment, and vasculopathy. [4,5,6,7] Juvenile xanthogranulomas and nevus anemicus are present in 80% of NF1 patients under the age of two. Therefore, these clinical features appear to be useful to improve early NF1 diagnosis in infants who may not present enough diagnosis criteria at their early age. [8]

This study aims to provide a literature review on the oral and cutaneous manifestations linked to NF1 and to present a clinical case of a NF1 patient who presents cutaneous and oral lesions.

## 2. Background

Manifestations of NF1, and especially cutaneous, oral, and cranio-facial alterations, are detailed in Table 1.

Cutaneous manifestations of NF1 are hallmarks of NF1 and are part of the diagnosis criteria. They consist of café au lait spots, freckles, and neurofibromas.

Café au lait spots are smooth-edged pigmented macules and their color varies from yellowish to chocolate-brown. They can appear anywhere on the skin; however, they are less common and numerous on the face [9]. Café au lait spots are present in 75% of children of 10 years affected by NF1 [6] and in 90% of NF1 adolescents and adults [9].

Freckles occur mostly in intertriginous regions such as axillary and inguinal areas and are referred to as Crowe’s sign. However, freckling may also occur diffusely over the trunk, extremities, upper eyelids, and base of the neck [9]. Freckling occurs in 80% of NF1 patients of 6 years or older which frequently allows diagnosis of young patients [10].

Neurofibromas are benign tumors and constitute one of the main characteristics of NF1. They are classified into two subtypes: localized and plexiform [10,11].

Localized neurofibromas arise from a single site along a peripheral nerve and appear as well-defined sessile or pedicled soft nodules which can be invoked by pressure (“button-hole sign”) [9,12]. They are mostly localized on the skin but can also arise on oral and vaginal mucosa. Some of them may also occur in deeper peripheral nerves, involving organs such as stomach, intestines, kidney, bladder, larynx, and heart. Regarding the head and neck area, scalp, cheek, neck, and oral cavity are the most affected regions [10,11]. Localized neurofibromas usually appear first during puberty and their number increase with age [1,3,13]. 84% of adults diagnosed with NF1 present localized neurofibromas [12]. However, these are not specific to NF1 as sporadic neurofibromas can occur without association to NF1 [14].

Plexiform neurofibroma is a poorly circumscribed and locally invasive tumor mass which extends along the length of a nerve trunk growing around distorted nerve fascicules and may spread along adjacent nervous rami, muscles, and skin [9,12,13,15]. These lesions may occur superficially or deeper inside the whole body and are a high source of morbidity as they grow to reach a great size, often producing disfigurement [9,13]. Plexiform neurofibromas appear at birth until 5 years of age and grow during childhood and adolescence [1], and then begin to decrease their growth potential with increasing age [16]. Sixteen to twenty-five percent of NF1 patients present with plexiform neurofibroma [12] which is pathognomonic of NF1 and does not occur sporadically [15].

The histopathological analysis of neurofibromas reveals a mixture of cell types including Schwann cells, perineurial cells, and fibroblasts. Schwann cells account for 40–80% of the cells and present with widened nuclei, undulated shape, and sharp corners [2,13]. Immunochemistry is fundamental to demonstrate the presence of neural involvement in these tumors through specific immunoreactions including S100, type IV collagen, CD34, and neurofilament or neuron specific tubulin TUBB3 [3].

Oral and cranio-facial manifestations of NF1 are increasingly highlighted in the literature, especially in regard to intra-oral alterations whose prevalence is estimated to be around 70% in regard to recent studies [2,4,6,11,16], with no predilection for gender or race [11].

Oral soft tissue alterations in relation to NF1 appear as prominent lingual papillae in 50% of cases [2,9] and mucosal and gingival neurofibromas in 25% of cases [2,4,11,13]. The preferential area where oral neurofibromas arise is the tongue [9], followed by buccal mucosa, lips, and gingiva [17]. Less frequently they can also affect the palate, the floor of the mouth, the major salivary glands, and the pharynx [5,16,17]. Macroglossia has been described in relation to plexiform neurofibromas arising inside the tongue [9]. In rare cases, patients with NF1 may present melanin pigmentation of their gingiva [4]. Neurofibromas arising on the oral cavity are mostly involved with NF1 [6], however, sporadic neurofibromas unassociated with NF1 can also occur at the rate of 6.5% [14,17]. Moreover, independently of their association with NF1, oral neurofibromas exhibit similar histological features as neurofibromas occurring in other sites [14].

Regarding cranio-facial alterations, orbital and sphenoidal wings dysplasia [3,4,16] are pathognomonic of NF1 and may induce exophthalmia [9].

Intra-osseus jaw bone lesions related to NF1 are also reported and consist mostly of the widening of the mandibular canal which may be associated with an irregular border of the canal and enlarged mandibular foramina unrelated to any tumor mass [3,4,7,9,16,18]. Intra-osseus neurofibromas resulting in well-defined radiolucent lesions have also been reported [7,9]. Moreover, NF1 patients may present with a retrognathic profile as they are more prone to presenting underdeveloped maxillaries and mandibles [3,7]. Other jaws deformities present in NF1 patients can be caused by local destruction or invasion of a plexiform neurofibroma [7]. Other maxillo-mandibular lesions including temporo-mandibular joint alterations have been reported, although less frequently, and will only be mentioned in Table 1.

Dental abnormalities are mostly linked to jaw alteration and/or gingiva lesions. Visnapuu et al. showed that dental age was unaffected in NF1 patients until the age of 17 years [3]. Retained or displaced teeth, as well as impaired growth of alveolar bone can occur in NF1 patients in association with gingival or bone neurofibromas and especially plexiform neurofibromas arising from the trigeminal nerve [4,7,13,16,19]. If a tumor affects the eruption of a tooth, surgical excision might allow its eruption [14].

## 3. Case Report

We report a case of a 51-year-old man diagnosed with NF1, addressed to our consultation for oral pain in his wisdom tooth 48. During anamnesis, the patient informed us about cutaneous café au lait spots since childhood, and that at puberty cutaneous neurofibromas located on the head, abdomen, back, and arms appeared, followed by the occurrence of oral neurofibromas around the age of 20. NF1 was then diagnosed and multiple cerebral MRI were carried out without revealing any lesion. Furthermore, he related having been diagnosed with idiopathic thrombocytopenia during childhood which required high dose corticosteroid treatment leading to side effects such as weight gain and increased hairiness. At the age of 31 years, he sustained surgical excision of multiple neurofibromas in the head and neck region in France. He also mentioned a tibial fracture which occurred five years earlier while skiing which led to an osteoporosis diagnosis, treated by intra-venous denosumab monoclonal antibody (Prolia) twice a year since then.

**Figure 1:**
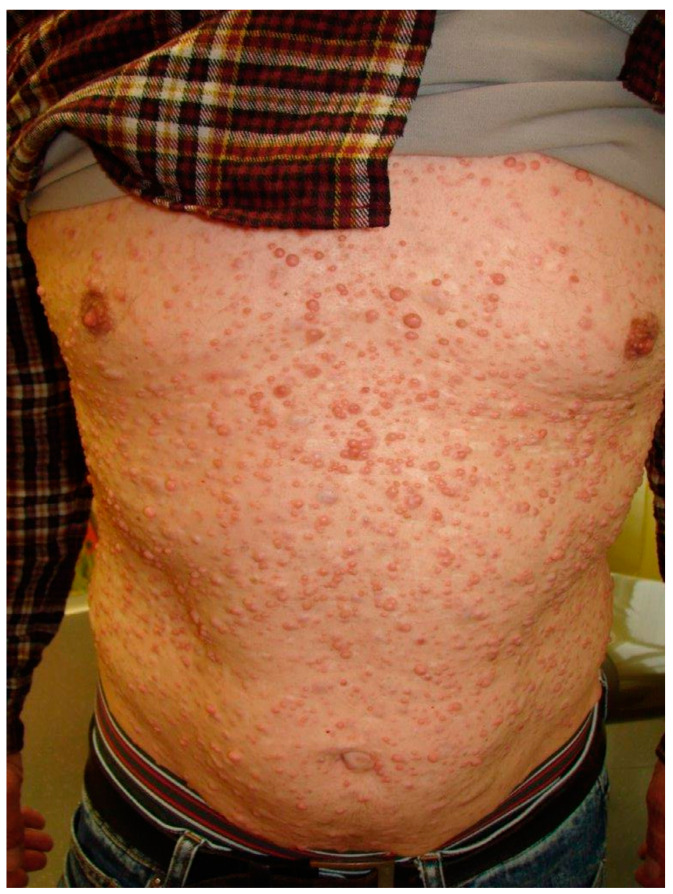
Multiplicity of cutaneous neurofibromas all over the abdomen and thorax.

**Figure 2:**
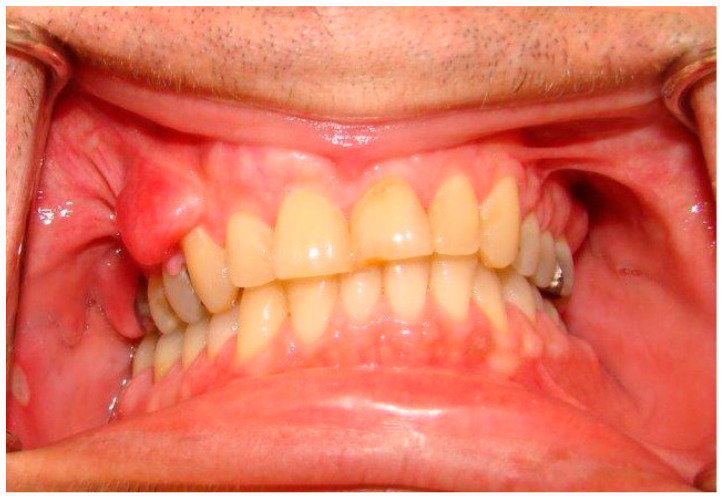
Presence of two oral neurofibromas on the right vestibular maxillary gingiva. Note also a round ulcer surrounded by an erythematous area on the right buccal mucosa, characteristic of an aphthous ulcer.

The clinical examination revealed numerous cutaneous neurofibromas on the neck and back of the head as well as on the abdomen and the back, arising to the gluteal fold (Figure 1). Apart from the infection related to the tooth 48, the intra-oral examination disclosed vestibular gingival nodules on the right side, measuring about one centimeter in diameter, covered by normal mucosa; the anterior one had a round aspect in comparison to the posterior one which had an elongated appearance most certainly due to the dental occlusion (Figure 2). The patient had however, no complaint in regard of those intra-oral nodules. A facial cone beam CT was performed and revealed no intra-osseus lesions linked to NF1.

**Figure 3:**
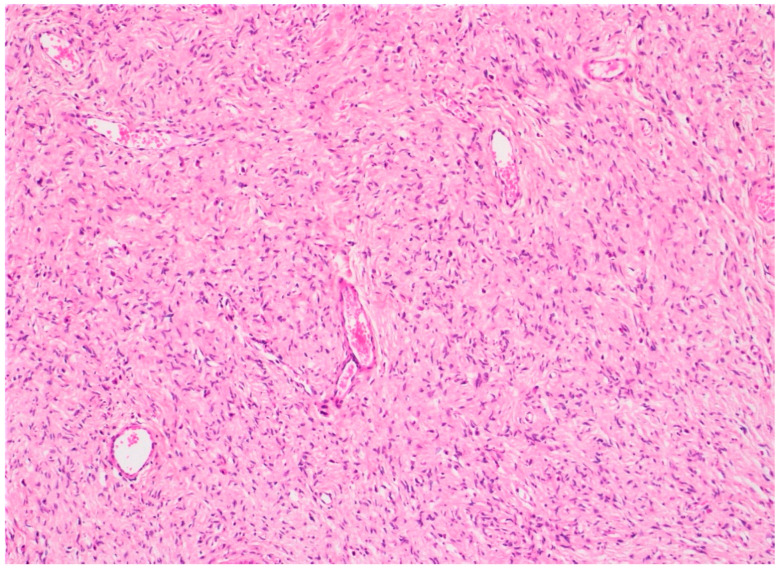
Histopathological section showing spindle cells with widened nuclei and undulated shape (HE stain, ×10).

**Figure 4:**
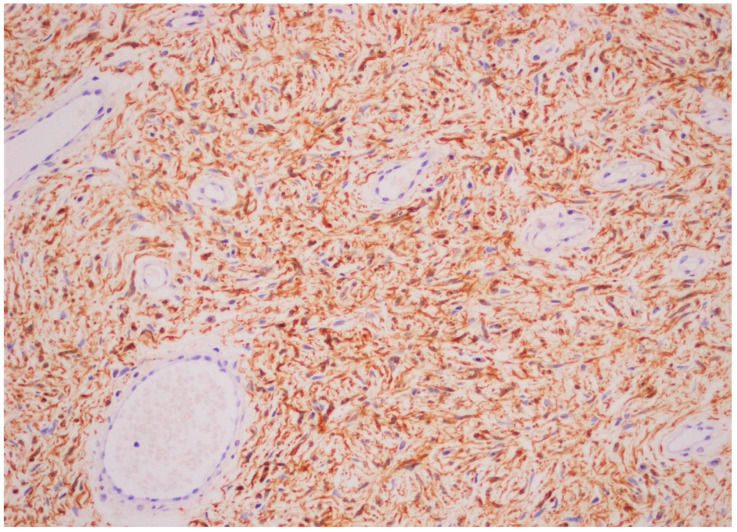
S-100 immunohistochemistry showing strong and diffuse positive stained cells (×20).

Tooth 48 was removed. However, its infection was not in relation with NF1. Two excisional biopsies of the maxillary lesions were also performed to assess a diagnosis and to allow the patient to have better oral hygiene. The histopathological analysis confirmed the diagnosis of oral neurofibromas in the context of NF1 (Figure 3 and Figure 4). The postoperative course was uneventful. Follow-up 11 years later showed no recurrence of the intra-oral lesions as well as no new lesions.

## 4. Discussion

NF1 is one of the only genetic disorders whose diagnosis is predominantly based on clinical criteria assessed in 1988 by Strumpf et al. To be diagnosed with NF1, the patients should fulfill two or more of the following criteria: six or more café au lait spots with a diameter ≥ 5 mm in prepubertal individuals or a diameter ≥ 15 mm in post-pubertal individuals; two or more neurofibromas of any type or one plexiform neurofibroma; axillary or inguinal freckling; optic glioma; one or more Lisch nodules of the iris; a distinct osseus lesion such as sphenoid dysplasia or pseudoarthrosis; and a first degree relative with NF1 according to the preceding criteria. [20] However, molecular diagnosis is frequently used to distinguish NF1 from Legius Syndrome/SPRED1 mutation and other rasopathies. Moreover, it allows NF1 diagnosis in case of a mosaic presentation [21].

This review highlights the high frequency of oral manifestations, especially neurofibromas, in addition to cutaneous lesions in NF1. It is only recently that these oral manifestations linked to NF1 have been highlighted in the literature as shown by their increased prevalence from 4–7% to 66–72%. [16]

The high prevalence of oral manifestations of NF1 should make clinicians attentive to look for these lesions and link them to NF1, as most oral neurofibromas only involve soft tissue and are not located intraosseous [17].

The clinical appearance of neurofibromas does not present with a specific aspect and remains similar to many other mucosal lesions of the oral cavity. Many other conditions can be evoked, such as epulis, ossifying fibroma, giant cell granuloma, odontogenic fibroma, and pyogenic granuloma for lesion occurring on the gingiva and salivary pseudocyst, hemangioma, lipoma, and dermoid cyst for those presenting on the oral mucosa. Excisional (or incisional) biopsies are therefore necessary to establish a definitive and accurate diagnosis. Furthermore, their excision may be assessed if they cause discomfort to the patient or prevent good oral hygiene.

The role of anamnesis and clinical examination take on their full meaning in order to establish the circumstances linked to the onset of the lesion and to assess if any similar lesion is present elsewhere on the skin or in another family member.

Neurofibromas present a risk of malignant degeneration, which is higher in case of plexiform neurofibromas (1–29%) [3,4,5] compared to localized neurofibromas (3–5%) [9,13]. The lifetime risk for NF1 patients to develop a malignant transformation from neurofibromas is evaluated between 8% and 13% [4,19].

Moreover, NF1 patients present a higher lifetime cancer risk (59.6%) in contrast to the general population (30.8%) [22]. Malignant peripheral nerve sheath tumor (MPNST) is the main malignancy which develops in 5% to 13% of NF1 patients [4,18,22], with poor prognosis [23]. Intra-cranial malignancies such as gliomas, ependymoma, and childhood astrocytomas are also well known [12,23]. Other malignant tumors such as breast cancer, gastrointestinal stromal tumor, thyroid cancer, rhabdomyosarcoma, malignant fibrous histiocytoma, pheochromocytoma, Wilms tumor, nonlymphocytic childhood leukemia [12,22], and exceptionally an intra-oral squamous cell carcinoma can also be encountered [24]. All malignant tumors arising in NF1 patients have a poor survival rate in comparison to matched controls [22].

Until recently, neurofibromas management consisted only in surgical resection, associated with a high hemorrhagic risk, depending on functional and/or esthetic demands of the patients, or in case of malignant transformation [1,13]. The emergence of the oral selective mitogen-activated protein kinase inhibitor selumetinib has now become the current standard of care for problematic plexiform neurofibroma, allowing their shrinkage in 68% of children [25].

In addition, regarding pediatric cases, it is well established that early intervention prevents significant deformity or organ compromise.

The general management of NF1 patients is multidisciplinary and mostly based on annual surveillance. It is of high importance that clinicians and especially dermatologists be acquainted with oral alterations in NF1 patients and include intra-oral examination during these follow-up appointments. Usually, these oral lesions are easily diagnosed in the context of NF1. In case of the presence of problematic oral lesion(s), clinicians should address these patients to a specific oral pathology consultation or proceed with an excisional biopsy.

## 5. Conclusions

Type I neurofibromatosis (NF1) is one of the most common genetic diseases and, given its genetic characteristic, affects individuals at a young age. Oral manifestations in relation to NF1 have been known for a long time, but their occurrence frequency was underestimated for a long period until recent studies increased their prevalence to around 70% of all NF1 individuals [2,4,6,10,15]. This article aimed to highlight the importance of oral examination when NF1 is suspected and to discuss neurofibromas as a potential differential diagnosis of oral nodules.

## Figures and Tables

**Table 1:** Cutaneous, oral, and cranio-facial manifestations linked to neurofibromatosis type 1 (NF1) [2,3,4,5,6,7,9,13,16,17,18,19].

**Cutaneous**	Café au lait spots *, axillary or inguinal freckling * (Crowe’s sign), cutaneous neurofibromas * (localized or plexiform).
**Oral soft tissue**	Prominent lingual papillae (50% cases); Mucosal and gingival neurofibromas * (25% of cases): mostly the tongue, followed by buccal mucosa, lips, and gingiva, and less commonly the palate, the floor of the mouth, the major salivary glands and the pharynx; Macroglossia in relation to plexiform neurofibromas arising inside the tongue; Melanin pigmentation of the gingiva (rare); Gingivitis or periodontitis in relation to oral neurofibromas prohibiting a proper oral hygiene.
**Cranio-facial**	Orbital dysplasia (may lead to exophthalmia), sphenoidal wings dysplasia *; Widening of the mandibular canal without relation with any tumor mass +/− irregular border of the canal and enlarged mandibular foramina; Short mandibular body, ramus, and condyle, undergrowth maxilla with hypoplasia of the maxillary tuberosity and short cranial base (inducing retrognathia); Intra-osseus neurofibromas of the maxilla/mandible and the temporo-mandibular joint (well-defined unilocular and occasionally multilocular radiolucent lesions); Notching of the posterior border of the mandibular ramus, elongated coronoid process with a deep sigmoid notch, hypoplasia of the condyle and zygomatic processes; Periapical cement dysplasia (only NF1 females affected), central giant cell granuloma, and osteolytic bone lesions linked to cherubism.
**Dental**	Retained or displaced teeth, agenesia, or hyperdontia, impaired growth of alveolar bone in association with gingival or bone neurofibromas and especially plexiform neurofibromas arising from the trigeminal nerve; Enamel defects such as molar-incisor hypomineralization, enamel hypoplasia, or opacities; Predisposition to caries is controversial.

* = part of diagnosis criteria of NF1.

## Data Availability

The data are not publicly available due to patient confidentiality and absence of electronic medical record (EMR) system.
